# Analysis of Cone-Beam Artifacts in off-Centered Circular CT for Four Reconstruction Methods

**DOI:** 10.1155/IJBI/2006/80421

**Published:** 2006-08-13

**Authors:** S. Valton, F. Peyrin, D. Sappey-Marinier

**Affiliations:** ^1^ CREATIS, UMR CNRS 5515, Villeurbanne 69621, France; ^2^ CREATIS, U630 INSERM, Villeurbanne 69621, France; ^3^ CREATIS, INSA-Lyon, Villeurbanne 69621, France; ^4^ CREATIS, Université Claude Bernard-Lyon1, Bron 69677, France

## Abstract

Cone-beam (CB) acquisition is increasingly used for truly three-dimensional X-ray computerized tomography (CT). However, tomographic reconstruction from data collected along a circular trajectory with the popular Feldkamp algorithm is known to produce the so-called CB artifacts. These artifacts result from the incompleteness of the source trajectory and the resulting missing data in the Radon space increasing with the distance to the plane containing the source orbit. In the context of the development of integrated PET/CT microscanners, we introduced a novel off-centered circular CT cone-beam geometry. We proposed a generalized Feldkamp formula (α-FDK) adapted to this geometry, but reconstructions suffer from increased CB artifacts. In this paper, we evaluate and compare four different reconstruction methods for correcting CB artifacts in off-centered geometry. We consider the α-FDK algorithm, the shift-variant FBP method derived from the T-FDK, an FBP method based on the Grangeat formula, and an iterative algebraic method (SART). The results show that the low contrast artifacts can be efficiently corrected by the shift-variant method and the SART method to achieve good quality images at the expense of increased computation time, but the geometrical deformations are still not compensated for by these techniques.


Hindawi Publishing Corporation
International Journal of Biomedical Imaging
Volume 2006, Article ID 80421, Pages 1–8
DOI 10.1155/IJBI/2006/80421

### Analysis of Cone-Beam Artifacts in off-Centered Circular CT for Four Reconstruction Methods

**S. Valton,[1,2,3,4] F. Peyrin,[1,2,3,4] and D. Sappey-Marinier[1,2,3,4]**

[1] *CREATIS, UMR CNRS 5515, 69621 Villeurbanne, France*
[2] *CREATIS, U630 INSERM, 69621 Villeurbanne, France*
[3] *CREATIS, INSA-Lyon, 69621 Villeurbanne, France*
[4] *CREATIS, Université Claude Bernard-Lyon1, 69677 Bron, France*

Received 1 December 2005; Revised 30 May 2006; Accepted 31 May 2006

Cone-beam (CB) acquisition is increasingly used for truly three-dimensional X-ray computerized tomography (CT). However, tomographic reconstruction from data collected along a circular trajectory with the popular Feldkamp algorithm is known to produce the so-called CB artifacts. These artifacts result from the incompleteness of the source trajectory and the resulting missing data in the Radon space increasing with the distance to the plane containing the source orbit. In the context of the development of integrated PET/CT microscanners, we introduced a novel off-centered circular CT cone-beam geometry. We proposed a generalized Feldkamp formula ($\alpha$-FDK) adapted to this geometry, but reconstructions suffer from increased CB artifacts. In this paper, we evaluate and compare four different reconstruction methods for correcting CB artifacts in off-centered geometry. We consider the $\alpha$-FDK algorithm, the shift-variant FBP method derived from the T-FDK, an FBP method based on the Grangeat formula, and an iterative algebraic method (SART). The results show that the low contrast artifacts can be efficiently corrected by the shift-variant method and the SART method to achieve good quality images at the expense of increased computation time, but the geometrical deformations are still not compensated for by these techniques.

Copyright © 2006 S. Valton et al. This is an open access article distributed under the Creative Commons Attribution License, which permits unrestricted use, distribution, and reproduction in any medium, provided the original work is properly cited.

### 1. INTRODUCTION

Tomographic reconstruction has been a very active research field for over twenty years. Early works were rather focused on the inverse problem of tomography [1], the theoretical basis of which was founded on the Radon inversion formula, while later contributions have mostly been devoted to practical reconstruction methods for specific acquisition geometries. Tomographic reconstruction methods are generally classified into two classes of reconstruction algorithms (see, e.g., [2, 3]): analytical and algebraic methods. The discretization of continuous inversion formulae leads to analytical methods while algebraic methods are based on the resolution of a linear system modeling the projection process. In algebraic reconstruction, the choice of the resolution technique results in a given algorithm (ART [4], SIRT [5], SART [6], etc.) characterized by an iteration formula which is completely independent of the acquisition geometry. In contrast, analytical methods are strongly dependent on the scanner geometry. Successive generations of scanners led to two-dimensional (2D) tomography with parallel-beam (PB) geometry, fan-beam (FB) geometry [7, 8], and today 3D cone-beam (CB) tomography [9–11]. 3D tomography enables the reconstruction of larger (multislice) volumes with reduced acquisition duration and irradiation.

The trajectory of the X-ray source is an important feature in 3D CB tomography. In addition to simple circle, numerous trajectories such as circle plus line [12], two circles [13], set of lines [14], helix, and so forth, have been investigated but currently, most contributions deal with the circular and the helical trajectories [15, 16]. The completeness condition on the source trajectory establishes whether or not exact reconstruction can be achieved from the projection data. According to the condition due to Kirillov [17] and formulated by Tuy [18] and Smith [19], a trajectory is complete if any plane crossing the support of the object intersects the vertex path at least once. No exact reconstruction can be expected if the source trajectory does not fulfill this condition owing to the incomplete projection data set.

In regular circular CB geometry, the most commonly used reconstruction algorithm is the FDK [20] which is a generalization to 3D CB of the standard filtered backprojection (FBP) algorithm. It is clear that the circular trajectory is incomplete since a plane parallel to the source path might

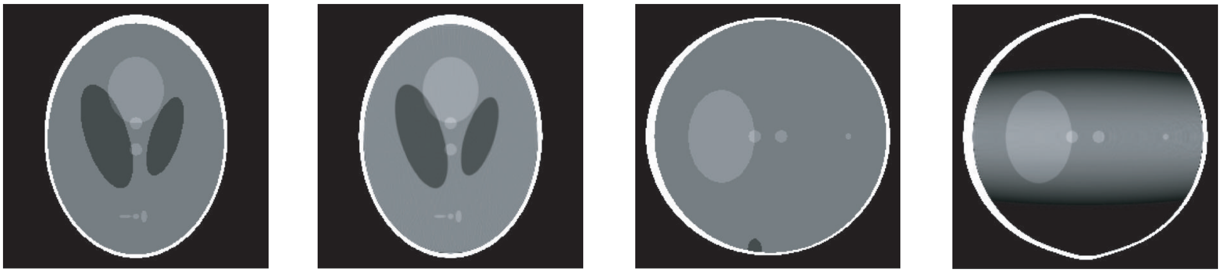

Figure 1: 3D Shepp-Logan phantom with total CB angle 36°. From left to right: horizontal mid-plane of the reference phantom, horizontal mid-plane of the reconstruction with the FDK algorithm, vertical plane of the reference phantom, and vertical plane of the FDK reconstruction.

intersect the support of the reconstructed volume while not crossing the source vertex. Reconstruction is therefore approximate except in the mid-plane (where it is equivalent to FB reconstruction). If reconstructions of good quality can be obtained with FDK for limited CB angles, artifacts rapidly increase with the distance to the mid-plane for a given focus length. Figure 1 represents the mid-plane and a vertical slice of a 3D Shepp-Logan phantom reconstructed with a total CB angle equal to 36°. The reconstruction suffers from two kinds of CB artifacts: drop of low contrast intensity and geometrical deformations which appear at contrast jumps along the rotation axis (here: vertical axis). In order to reduce CB artifacts, different approaches have been reported in the past few years. Grass et al. [21] proposed the T-FDK algorithm which relies on changing the ramp filtering direction through CB to parallel fan-beam (PFB) rebinning. Yu et al. [22] have improved the reconstruction resolution via shift-variant filtering. Another class of methods is based on the Grangeat formula which relates the CB projections to the derivative of the 3D Radon transform [23]. The Radon transform reconstituted from the CB projection collected along a circular trajectory forms a torus instead of a sphere. This difference, referred to as the shadow zone, corresponds to the missing data. The reconstruction obtained with zero padding of the shadow was presumed to be equivalent to FDK and therefore suffered from CB artifacts. Lee et al. showed that filling in the shadow zone by interpolation could improve reconstruction [24]. Moreover, Hu made an important contribution providing a link between the Grangeat and the FDK formulae. He established that the FDK formula only deals with the inner part of the Radon torus and proposed a method which added the contribution of the Radon data on the torus shell [25]. Yang et al. completed this approach with information on the shadow zone [26]. More empirical approaches were suggested such as a 3D weighting of the projection data before the backprojection step [23] or error-correction-based methods [27–29] which associate analytical and algebraic approaches.

We recently introduced a novel acquisition geometry for CT data which enables simultaneous acquisition of PET and CT data for small animal [30]. This concept is based on photon counting pixel X-ray detectors expected to have a very high counting rate at X-rays energy (around 50 keV) while not stopping gamma rays (511 keV) [31]. This feature permits their placement inside a full micro-PET ring, in front of the PET crystals. However, this configuration imposes the X-ray source to be located outside the PET ring on an independently rotating system and focused on the center of the PET field of view (FOV). In this design, the X-ray source is off-centered with respect to the volume of interest (VOI) which results in the rotation of the source and the detector in two distinct planes. We first proposed a generalized formula called $\alpha$-FDK [30] allowing for this topology, which comes down to standard FDK when applied to the simple circular geometry. However, the off-centered geometry is clearly exposed to CB artifacts since the entire volume of interest might be outside the exactly reconstructed mid-plane. The $\alpha$-FDK performs the reconstruction of a shifted (or not) VOI defined by the angle $\alpha$ and partially compensates for the low-contrast attenuation via the addition of an offset to obtain the correct value in the central plane. Nevertheless, the formula produces geometrical deformations and does not suppress the ramp of the reconstructed intensity. Reconstruction results obtained on simulations with the $\alpha$-FDK therefore suffered from severe CB artifacts with large values of $\alpha$ and multislice volumes.

The purpose of this paper is to evaluate four different reconstruction techniques in our specific off-centered geometry in terms of CB artifacts reduction. We consider three analytical reconstruction methods, the $\alpha$-FDK [30], the adaptation to our geometry of the shift-variant filtering (SVF) technique [22], and an FBP Grangeat-based (GB) formula [25]. We also evaluate an algebraic method, SART [6].

The paper is organized as follows. Section 2 gives a detailed description of the acquisition geometry. The four reconstruction methods are presented in Section 3. The reconstruction results obtained by computer simulations are presented and discussed in Section 4.

### 2. ACQUISITION GEOMETRY

The off-centered geometry parameterized by angle $\alpha$ is depicted in Figure 2. Let $f(x, y, z)$ be a 3D attenuation function whose support is a sphere of radius $r$ centered on the origin $O$ of a Cartesian coordinate system. The plane $(O, x, y)$ is called the central plane. The X-ray source is located at a

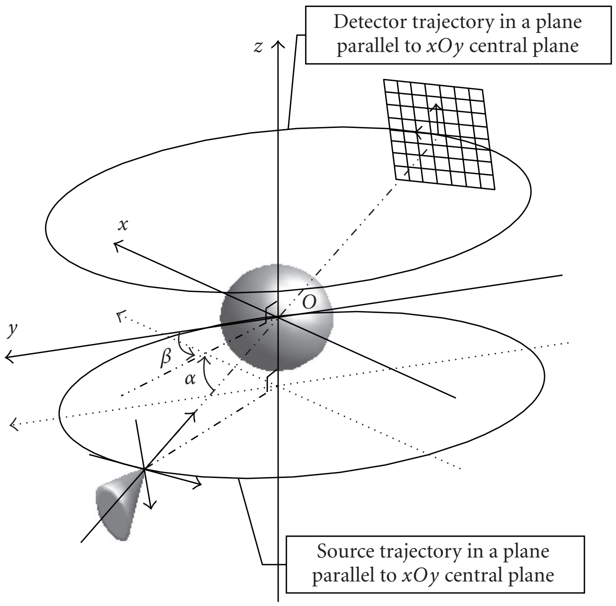

FIGURE 2: Off-centered geometry: the X-ray source (depicted by the small cone) and the detector rotate in two parallel planes, the offset of which is parameterized by angle $\alpha$. The position of the source at each projection is given by angle $\beta$.

distance $R$ from the origin where $R > r$ and rotates in the plane $z = c$ where $c \neq 0$, called the mid-plane. The X-ray tube is oriented so that the central ray passes through $O$. Its position on the vertex path is parameterized by angle $\beta$. The acquisition geometry is characterized by $\alpha$, defined as the angle between the central ray and the central plane. Detector plane is perpendicular to the central ray and faces the source at a distance $D$. For the sake of simplicity and without loss of generality, we will consider a virtual detector whose center is on the $z$ axis, that is, $D = R$. Let $(u, v)$ be the coordinate system centered on the orthogonal projection of the source on the detector, where $u$ refers to the lines of the detector ($z$ constant) and $v$ refers to the columns. Let $p_{\alpha,\beta}(u, v)$ denote the 2D off-centered CB projection at angular position $\beta$ in the off-centered geometry defined by angle $\alpha$.

### 3. RECONSTRUCTION METHODS

### 3.1. $\alpha$-FDK

We derived a modified FDK expression adapted to the parameterization of the off-centered geometry. We recall the generalized inversion $\alpha$-FDK formula [30]

$$f_{\alpha\text{-FDK}}(x, y, z) = \int_0^{2\pi} \frac{1}{U^2} \widetilde{p}_{\alpha,\beta}\left(\frac{t'}{U}\frac{r'}{U}\right) d\beta, \tag{1}$$

where the weight $U$ is given by

$$U = \frac{D - s'}{D} \tag{2}$$

and $(t', s', r')$ is the coordinates system obtained by rotation of angle $\beta$ around $z$ axis followed by rotation of angle $-\alpha$ around $x$ axis so that the $s'$ axis points toward the source. Coordinates $t'$, $s'$, and $r'$ are given by

$$\begin{aligned} t' &= x\cos\beta + y\sin\beta, \\ s' &= -x\cos\alpha\sin\beta + y\cos\alpha\cos\beta - z\sin\alpha, \\ r' &= x\sin\alpha\sin\beta - y\sin\alpha\cos\beta + z\cos\alpha. \end{aligned} \tag{3}$$

The preweighting and filtering operations are given by

$$\begin{aligned} p'_{\alpha,\beta}(u, v) &= \frac{D}{\sqrt{D^2 + u^2 + v^2}} p_{\alpha,\beta}(u, v), \\ \widetilde{p}_{\alpha,\beta}(u, v) &= p'_{\alpha,\beta}(u, v) * \frac{1}{2} h(u), \end{aligned} \tag{4}$$

where $h$ is the ramp filter.

This formula is equivalent to the standard FDK when $\alpha = 0$. From a practical point of view, the $\alpha$-FDK offers the same advantages as FDK for implementation: filtering is made on the lines of the detector, and each projection can be processed independently making the algorithm easily parallelizable.

### 3.2. *T-FDK with shift-variant filtration*

The algorithm proposed in [22] by Yu et al. for CB reconstruction is an FBP applied to parallel fan-beam data. The CB-to-PFB rebinning is performed via the Fourier space. The rebinned PFB projections are given by pr′ $\varphi(u, v)$ where angle $\varphi$ satisfies

$$\varphi = \beta - \arctan\left(\frac{u}{R}\right). \tag{5}$$

The projections obtained after the CB-to-PFB rebinning operation lie on a curved virtual detector where the native coordinates are no longer equispaced. This step is thus followed by a vertical rebinning to obtain vertically equispaced $\mathrm{pr}_{\varphi}(u, ve)$ projection data where

$$ve = v\frac{R^2}{R^2 + u^2}. \tag{6}$$

Then, a similar horizontal rebinning is performed in [21] while Yu et al. alternatively propose to modify the 1D ramp filter. A shift-variant filtering of the nonrebinned data is applied instead of the ramp filtering of the equispaced data. Let $\xi$ be the equispaced coordinate along the detector lines given by

$$\xi = \frac{uR}{\sqrt{u^2 + R^2}}. \tag{7}$$

Then one obtains via a changing variable the following reconstruction formula:

$$\begin{aligned} &f_{\mathrm{SVF}}(x, y, z) \\ &\quad = \frac{1}{2}\int_0^{2\pi} d\varphi \int_{-u_{\max}}^{u_{\max}} D(u)\,\mathrm{pr}_{\varphi}\,(u, ve_0)\,G(u_0, u)\,h(u_0 - u)\,du, \end{aligned} \tag{8}$$

where

$$ve_0(x,y,z,\varphi) = \frac{z\sqrt{R^2-\xi^2}}{\sqrt{R^2-\xi^2}+x\sin\varphi - y\cos\varphi},$$

$$D(u) = \frac{d\xi}{du} = \left(\frac{R}{\sqrt{u^2+R^2}}\right)^3, \quad (9)$$

$$G(u_0,u) = \left(\frac{u_0-u}{Ru_0/\sqrt{R^2+u_0{}^2} - Ru/\sqrt{R^2+u^2}}\right).$$

This formula has two drawbacks compared to the standard FDK:

(i) the CB-to-PFB rebinning requires all the CB projections which is memory and time consuming,

(ii) the shift-variant filter kernel prevents from implementing the filtering in the Fourier space.

### 3.3. Radon-based method (RB)

The 3D Radon transform $\Re f(\rho,\vec{n})$ of the function $f$ corresponds to its integral along the plane of normal vector $\vec{n}$ at a distance $\rho$ from the origin:

$$\Re f(\rho,\vec{n}) = \iint_{M\in P(\rho,\vec{n})} f(M)dM. \quad (10)$$

Hu showed in [25] that the function $f_{\mathrm{RB}}$ reconstructed with the Grangeat formula based on the 3D Radon inversion could be expressed as the sum of three terms:

$$f_{\mathrm{RB}} = f_{M0} + f_{M1} + f_N. \quad (11)$$

The first term corresponds to the FDK formula, or the inversion of the 3D Radon data contained into the torus. The second term can be interpreted as the inversion of the 3D Radon data situated on the torus shell and was expressed as a filtered backprojection. The last term is a contribution from the shadow zone which is set to 0 in Hu's method.

The first term is thus given by

$$f_{M0} = f_{\mathrm{FDK}}. \quad (12)$$

The second term is expressed as

$$f_{M1}(x,y,z) = -\frac{1}{4\pi^2}\int_0^{2\pi}\frac{z}{R+x\sin\beta - y\cos\beta}\frac{\partial\sigma_\beta(\nu)}{\partial\nu}d\beta, \quad (13)$$

where the Radon data on the circle which describes the radon shell is obtained by

$$\sigma_\beta(\nu)\int_{u\min}^{u\max} p'_\beta(u,\nu)du, \quad (14)$$

where the preponderate projection $p'_\beta$ is given by (4) with $p'_\beta = p'_{\alpha=0,\beta}$.

It is important to note that both the RB and the SVF methods are given for standard trajectory. We therefore performed reconstructions with these methods by considering a shifted VOI to account for the off-centered geometry without modifying the algorithms.

### 3.4. SART

Algebraic methods are based on a discrete modeling of the reconstruction problem. The 3D attenuation function is represented by an $N$-element vector $\mathbf{F}$ and for each $\boldsymbol{\beta}$, the projections by an $M$-element vector $\mathbf{P}_{\alpha\beta}$ obtained by the following matrices product:

$$\mathbf{P}_{\alpha\beta} = \mathbf{R}_{\alpha\beta}\mathbf{F}, \quad (15)$$

where $\mathbf{R}_{\alpha\beta}$ is the $M\times N$ matrix describing the projection process. The purpose is to find $\mathbf{F}$, given projections $\mathbf{P}_{\alpha\beta}$ for $\boldsymbol{\beta}$ describing a discrete set of angles. The solution $\mathbf{F}$ is then approached by iterative corrections of the volume $\mathbf{F}^k$. The algorithm we implemented is the SART (simultaneous algebraic reconstruction technique) and adopts the following scheme.

For each cycle,

(i) for each projection, the following is required:

  (1) estimation of the projection of the reconstructed volume $F^k$,

  (2) difference between real projection and estimated projection,

  (3) normalization of the error,

  (4) backprojection of the error.

This algorithm is summarized by the iteration formula

$$F_n^{k+1} = F_n^k + \lambda\frac{\sum_{m=1}^{M}((\mathbf{P}_{\alpha\beta n} - \mathbf{R}_{\alpha\beta m}\mathbf{F}^k)/\sum_{n=1}^{N}R_{\alpha\beta mn})R_{\alpha\beta mn}}{\sum_{m=1}^{M}R_{\alpha\beta mn}}, \quad (16)$$

where $N$ is the number of voxels, $M$ the number of pixels on the projections, and $\lambda$ is a relaxation parameter. The SART algorithm was implemented on the basis of the $\alpha$-FDK, with minimum modifications. The projection matrices used for the backprojection were used reversely to compute the reprojections. A bilinear interpolation on the detector was used both for back- and reprojection, and the relaxation parameter $\lambda$ was set to 1. This implementation produces numerical artifacts which were smoothed by the application of a mean filter to the final reconstructed volume.

### 4. SIMULATION RESULTS

### 4.1. Simulation parameters

Simulations were performed with the 3D Shepp-Logan phantom [32]. The acquisition system was defined with the features given in Table 1 where all distances are given in centimeters.

We tested the reconstruction with six values of angle $\alpha$ between 0 and 0.5 radian which corresponds to 0°. to 28.5°. The resulting half-CB angle varies from 9.5° to 50° which is very large compared to current values in standard CB geometry.

The four methods developed in Section 3 were implemented and results were studied on simulations. The peak-to-peak signal-to-noise ratio (PPSNR) is used for the quantitative evaluation of the reconstructions. It was computed

Table 1: Simulation parameters.

| | | Detector | | Volume | | Number of projections |
|---|---|---|---|---|---|---|
| $R$ | $D$ | Number of pixels | Pixel side size | Number of voxels | Voxel side size | |
| 6 | 6 | 256 × 256 | 0.0078125 | 256 × 256 × 256 | 0.0078125 | 256 |

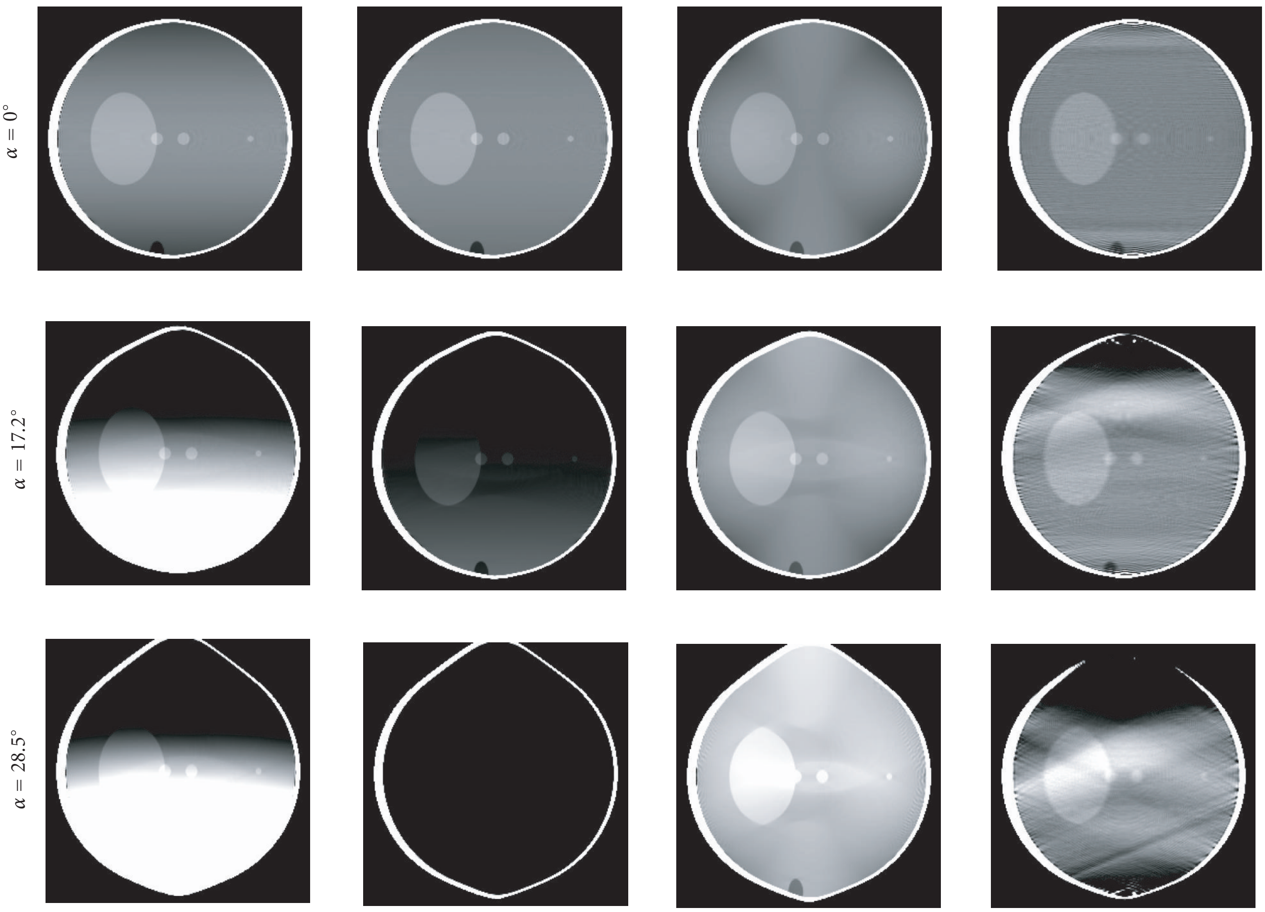

(a) (b) (c) (d)

Figure 3: Vertical slice of the 3D Shepp-Logan phantom reconstructed with: (a) $\alpha$-FDK, (b) RB, (c) SVF, and (d) SART. From top to bottom, the lines correspond to 3 values of $\alpha$ (from top to bottom): 0°, 17.2°, and 28.5°. The display window is [0.98, 1.06].

using the following expression:

$$10^* \log_{10}\left(\frac{\text{recostructed image range}^2}{\text{MSE}}\right), \qquad (17)$$

where MSE is the mean square error between the reconstructed and the reference phantom volume. In the following subsections, we present and discuss reconstruction results obtained with the three analytical methods and the SART algorithm after ten cycles.

### 4.2. Reconstruction results

Figure 3 displays vertical planes extracted from volumes reconstructed with the different algorithms for three values of $\alpha$: 0°, 17.2°, and 28.5°. The corresponding profiles along $z$-axis for $\alpha = 28.5°$ are plotted in Figure 4.

As expected, the volumes reconstructed with $\alpha$-FDK (Figure 3(a)) show two kinds of asymmetrical artifacts when $\alpha \neq 0$: vertical geometrical deformations and drop of low-contrast intensity. The vertical profiles displayed in Figure 4 include the result obtained with the standard FDK and reveal an offset between the two formulae. The RB algorithm (Figure 3(b)) partially compensates for the intensity drop but since no offset is used, the reconstructed intensity stays below the result of the $\alpha$-FDK (Figure 4). In Figure 3, the result with $\alpha = 28.5°$ appears dark because the same grey level window was used for comparison between the different reconstructions. The SVF algorithm (Figure 3(c)) better compensates

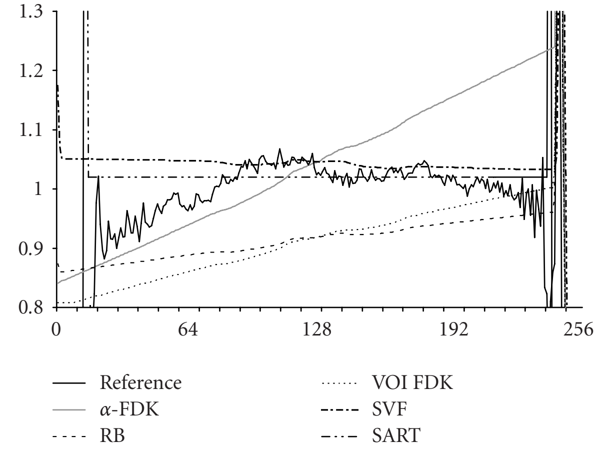

Figure 4: Vertical profile plot of the reconstructed Shepp-Logan at $\alpha = 28.5^\circ$.

for the intensity drop but the vertical elongation remains. The results obtained with standard SART after 10 cycles displayed in Figure 3(d) show that the intensity drop is only partially compensated for while the elongation is slightly corrected. The PPSNRs reported in Table 2 indicate that, quantitatively, the best reconstruction is obtained with the algebraic algorithm.

### 4.3. Discussion

In standard circular trajectory, CB artifacts correction is already a challenge since those artifacts come from the ill-conditioning of the inverse tomographic problem. In off-centered geometry, this problem is worse since the CB angle is more important and the VOI is asymmetrical with respect to the exactly reconstructed mid-plane.

The $\alpha$-FDK provides a practical formula adapted to the parameterization by the angle $\alpha$ of the off-centered geometry while preserving the rapidity and simplicity of the standard formulation. It compensates for the low-contrast drop by adding an offset to the reconstructed value. However, since it does not suppress the increase of the drop with the distance to the mid-plane, this compensation is fully valuable only for a small volume around the central plane of the VOI.

The RB method reduces the CB artifacts in standard circular geometry but the correction is weak. In off-centered geometry, the $\alpha$-FDK outperforms the RB method when $\alpha$ is large. In addition, the method presented by Hu takes 20% more time than the $\alpha$-FDK.

Concerning the SVF method, the simulation results presented above show that the intensity drop is well corrected by this method which, however, does not deal with the geometrical deformations. We can notice in Table 2 that the PPSNR is slightly inferior to that obtained with the $\alpha$-FDK until $\alpha = 28.5^\circ$. We presume that this difference is due to the numerical errors introduced by the different rebinning operations. Given the supplementary rebinning step and the shift-variant filtering, this algorithm is more time and memory consuming than the $\alpha$-FDK. Besides, the rebinning step prevents from starting the reconstruction before the end of the acquisitions. To conclude, reconstructions obtained with the SVF algorithm are better than those obtained with the $\alpha$-FDK, but the computation time is twice as long. The choice of the optimal reconstruction method thus depends on the constraints of the application. We have seen that the reconstruction obtained with the $\alpha$-FDK for a volume made of a limited number of slices is acceptable. Therefore, in the perspective of a bimodal scanner, it might be interesting to prefer this simple algorithm if the axial FOV of microPET scanners is limited to few slices, depending on the number of detector rings.

Our implementation of the SART algorithm produces numerical noise resulting from the model of forward projection and the value of the relaxation factor. This high-frequency noise appears very clearly in the picture if no mean filtering is applied because of the narrow grey level display window. In contrast, it has minor effects on the PPSNR thanks to its low amplitude and MSE. The high PPSNR compared to the other methods is related to the algebraic nature of the SART which minimizes a numerical error, namely, the difference between the real and the estimated projection. The profile plot in Figure 4 shows that the correction of intensity drop is less efficient than with the SVF method, but a close look at the right end of the graph points out that the elongation is slightly corrected by this algebraic reconstruction. Nevertheless, we think that this minor improvement is not worth the additional time expense needed to perform 10 cycles of the algorithm.

To our knowledge, no correction method compensates for the geometrical deformations due to the large CB angle. Actually, no clear mention of this kind of artifacts is made in the literature dealing with CB artifact since they clearly appear only with very large values of the CB angle. We believe that this aspect needs to be addressed in future works concerning CB artifacts reduction and that the correction of the elongation should, at least partially, compensate for the low-contrast drop.

### 5. CONCLUSION

In this paper, a comparison between the results obtained with four different reconstruction techniques on data simulated with an off-centered CB circular acquisition geometry was presented. Since this geometry increases the proportion of missing data compared to the standard circular trajectory, the CB artifacts correction methods developed for the standard case need to be reinvestigated. On the one hand, the $\alpha$-FDK reconstruction algorithm that we derived in a previous work produces strong CB artifacts, but a good correction is obtained in the planes closed to the VOI central plane, without additional time expense. On the other hand, the intensity drop is corrected by the RB algorithm, but not sufficiently enough, by the SART and even better by the SVF algorithm in return for a certain time expense. The preferable method therefore depends on the application, in terms of computation time and accuracy needs. Generally speaking, the SVF is

Table 2: Signal-to-noise ratio in dB obtained with the four reconstruction methods for increasing values of $\alpha$.

| Angle $\alpha$ | 0° | 5.7° | 11.5° | 17.2° | 22.9° | 28.5° |
|---|---|---|---|---|---|---|
| $\alpha$-FDK | 27.03 | 26.83 | 26.34 | 25.53 | 24.40 | 23.06 |
| RB | 26.89 | 26.75 | 25.89 | 24.99 | 24.32 | 22.72 |
| SVF | 26.29 | 26.29 | 25.65 | 24.94 | 24.37 | 23.08 |
| SART | 30.73 | 30.57 | 30.16 | 29.42 | 28.42 | 27.15 |

preferable to the SART owing to the expensive computation time of the algebraic method, and to the $\alpha$-FDK thanks to the attenuation correction. For limited number of slices and important value of $\alpha$, the $\alpha$-FDK gives better results than the RB method and can be used instead of SVF for its rapidity. Nonetheless, the geometrical deformations could not be efficiently corrected. Further research is therefore necessary to address this complicated problem inherent to CT.

### REFERENCES

[1] G. T. Herman, *Image Reconstruction from Projections*, chapter 14, Academic Press, New York, NY, USA, 1980.

[2] F. Natterer, "The Mathematics of Computerized Tomography," John Wiley & sons, Chichester, NY, USA, 1986.

[3] A. C. Kak and M. Slaney, *Principles of Computerized Tomographic Imaging*, IEEE Press, New York, NY, USA, 1987.

[4] R. Gordon, R. Bender, and G. T. Herman, "Algebraic reconstruction techniques (ART) for three-dimensional electron microscopy and x-ray photography," *Journal of Theoretical Biology*, vol. 29, no. 3, pp. 471–481, 1970.

[5] P. Gilbert, "Iterative methods for the three-dimensional reconstruction of an object from projections," *Journal of Theoretical Biology*, vol. 36, no. 1, pp. 105–117, 1972.

[6] A. H. Andersen and A. C. Kak, "Simultaneous algebraic reconstruction technique (SART): a superior implementation of the art algorithm," *Ultrasonic Imaging*, vol. 6, no. 1, pp. 81–94, 1984.

[7] G. T. Herman, A. V. A. Lakshminarayanan, and A. Naparstek, "Convolution reconstruction techniques for divergent beams," *Computers in Biology and Medicine*, vol. 6, no. 4, pp. 259–271, 1976.

[8] G. T. Gullberg, "The reconstruction of Fan Beam Data by filtering the back-projection," *Computer Graphics and Image Processing*, vol. 10, no. 1, pp. 30–47, 1979.

[9] C. Hamaker, K. T. Smith, D. C. Solmon, and S. L. Wagner, "The divergent beam X-ray transform," *Rocky Mountain Journal of Mathematics*, vol. 10, pp. 253–283, 1980.

[10] F. Peyrin, "The generalized back-projection theorem for cone-beam projection data," *IEEE Transactions on Nuclear Science*, vol. 32, pp. 1512–1219, 1985.

[11] B. D. Smith, "Cone-beam tomography. Recent advances and a tutorial review," *Optical Engineering*, vol. 29, no. 5, pp. 524–534, 1990.

[12] G. L. Zeng and G. T. Gullberg, "A cone-beam tomography algorithm for orthogonal circle-and-line orbit," *Physics in Medicine and Biology*, vol. 37, no. 3, pp. 563–577, 1992.

[13] H. Kudo and T. Saito, "Fast and stable cone-beam filtered backprojection method for non-planar orbits," *Physics in Medicine and Biology*, vol. 43, no. 4, pp. 747–760, 1998.

[14] G. Wang, T.-H. Lin, P. Cheng, and D. M. Shinozaki, "A general cone-beam reconstruction algorithm," *IEEE Transactions on Medical Imaging*, vol. 12, no. 3, pp. 486–496, 1993.

[15] A. Katsevich, "Theoretically exact filtered backprojection-type inversion algorithm for spiral CT," *SIAM Journal on Applied Mathematics*, vol. 62, no. 6, pp. 2012–2026, 2002.

[16] H. Kudo, T. Rodet, F. Noo, and M. Defrise, "Exact and approximate algorithms for helical cone-beam CT," *Physics in Medicine and Biology*, vol. 49, no. 13, pp. 2913–2931, 2004.

[17] A. A. Kirillov, "On a problem of IM Gel'fand," *Soviet Mathematics Doklady*, vol. 2, pp. 268–269, 1961, English Translation.

[18] H. K. Tuy, "An inversion formula for cone-beam reconstruction," *SIAM Journal on Applied Mathematic*, vol. 43, no. 3-4, pp. 546–552, 1983.

[19] B. D. Smith, "Cone-beam convolution formula," *Computers in Biology and Medicine*, vol. 13, no. 2, pp. 81–87, 1983.

[20] L. A. Feldkamp, L. C. Davis, and J. W. Kress, "Practical cone-beam algorithm," *Journal of the Optical Society of America A: Optics and Image Science, and Vision*, vol. 1, no. 6, pp. 612–619, 1984.

[21] M. Grass, Th. Köhler, and R. Proksa, "3D cone-beam CT reconstruction for circular trajectories," *Physics in Medicine and Biology*, vol. 45, no. 2, pp. 329–347, 2000.

[22] L. Yu, X. Pan, and C. A. Pelizzari, "Image reconstruction with a shift-variant filtration in circular cone-beam CT," *International Journal of Imaging Systems and Technology*, vol. 14, no. 5, pp. 213–221, 2004.

[23] P. Grangeat, "Mathematical framework of cone-beam 3D reconstruction via the first derivative of the Radon transform," in *Mathematical Methods in Tomography*, G. T. Herman, A. K. Louis, and F. Natterer, Eds., Lecture Notes in Mathematics, pp. 66–97, Springer, New York, NY, USA, 1991.

[24] S. W. Lee, G. Cho, and G. Wang, "Artifacts associated with implementation of the Grangeat formula," *Medical Physics*, vol. 29, no. 12, pp. 2871–2880, 2002.

[25] H. Hu, "An improved cone-beam reconstruction algorithm for the circular orbit," *Scanning*, vol. 18, pp. 572–581, 1996.

[26] H. Yang, M. Li, K. Koizumi, and H. Kudo, "A FBP-type cone-beam reconstruction algorithm with Radon space interpolation ability for axially truncated data from a circular orbit," in *Proceedings of the 8th International Conference on Fully 3D Reconstruction in Radiology and Nuclear Medicine*, pp. 401–404, Salt Lake City, Utah, USA, July 2005.

[27] J. Hsieh, "A practical cone-beam artifact correction algorithm," in *Poceedings of the IEEE Nuclear Science Symposium Conference Rcord (NSS '00)*, vol. 2, pp. 15/71–15/74, Lyon, France, October 2000.

[28] K. Zeng, Z. Chen, L. Zhang, and G. Wang, "A half-scan error reduction based algorithm for cone-beam CT," *Journal of X-Ray Science and Technology*, vol. 12, no. 2, pp. 73–82, 2004.

[29] K. Zeng, Z. Chen, L. Zhang, and G. Wang, "An error-reduction-based algorithm for cone-beam computed tomography," *Medical Physics*, vol. 31, no. 12, pp. 3206–3212, 2004.

[30] S. Valton, F. Peyrin, and D. Sappey-Marinier, "Generalization of 3D tomographic reconstruction algorithm FDK for an off-centred geometry," in *Proceedings of the IEEE International*

*Conference on Image Processing (ICIP '05)*, vol. 3, pp. 620–623, Genova, Italy, September 2005.

[31] P. Delpierre, S. Basolo, J. F. Berar, et al., "Pixscan: pixel detector CT-scanner for small animal imaging," in *Poceedings of the IEEE Nuclear Science Symposium Conference Rcord (NSS '05)*, vol. 4, pp. 2381–2385, San Juan, Puerto Rico, USA, October 2005.

[32] C. Axelsson, *Direct Fourier methods in 3D-reconstruction from cone-beam data*, Ph.D. thesis, Department of Electrical Engineering, Linköping University, Linköping, Sweden, 1994, p. 123.

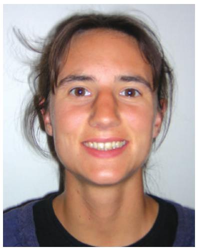

**S. Valton** was born in France in 1982. She received her M.S. degree in computer science from the University Jean Monnet in Saint Etienne and her Engineer degree from ISTASE, both in France in 2004. She is currently a Ph.D. student at the research center CREATIS, INSA-Lyon. Her research interest includes tomographic renconstruction.

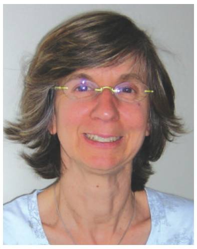

**F. Peyrin** received her Doctorate degree in computer science in 1982, and her "Docteur ès Sciences" degree, both from the University of Lyon and the National Institute of Applied Sciences (INSA-Lyon), France, respectively, in 1982 and 1990. Since 1981, she has been a Researcher in the CREATIS Laboratory at INSA-Lyon. Her research interests include 3D imaging techniques particularly in X-ray tomography, 3D image analysis, and wavelet-based methods. She is now Director of Research at INSERM and leads a group on "3D bone architecture analysis" in the laboratory, and she is currently a Scientific Collaborator at the ESRF, Grenoble, France.

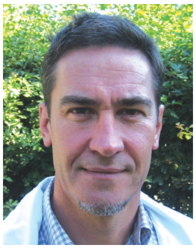

**D. Sappey-Marinier** received his Doctorate degree in biomedical engineering in 1987 from the University "Claude Bernard" of Lyon, France, and performed his postdoctorate fellowship at University of California, San Francisco, for 3 years. In 1993, he became an Associate Professor at the Medical School of the University "Claude Bernard" of Lyon, France. He has been a Researcher in the CREATIS Laboratory since 2003. His research activity concerns the development of functional and metabolic imaging techniques such as magnetic resonance, PET, and CT. He is currently coordinating the group of Lyon from the Crystal Clear European collaboration on the development of the ClearPET.

